# Ribosomal RNA operon copy number: A trait‐informed framework to close the microbial cultivation gap

**DOI:** 10.1002/imo2.70071

**Published:** 2025-12-25

**Authors:** Yuxiao Huang, Qingliu Wu, Chang Liu, Pugang Yu, Hao Li, Shengyue Tang, Fusuo Zhang, Shuangjiang Liu, Bin Ni

**Affiliations:** ^1^ State Key Laboratory of Nutrient Use and Management, College of Resources and Environmental Sciences, National Academy of Agriculture Green Development China Agricultural University Beijing China; ^2^ State Key Laboratory of Microbial Technology Shandong University Qingdao Shandong China; ^3^ State Key Laboratory of Microbial Diversity and Innovative Utilization, Institute of Microbiology Chinese Academy of Sciences Beijing China; ^4^ College of Life Sciences University of Chinese Academy of Sciences Beijing China

## Abstract

The vast majority of microbes remain uncultured, constraining functional validation and limiting microbiome‐based applications. We show that ribosomal RNA operon (*rrn*) copy number—a stable, phylogenetically conserved genomic trait—is strongly correlated with cultivation outcomes across ecosystems. Comparative analyses of rhizosphere, human gut, and mouse gut microbiomes reveal that rhizosphere communities contain a higher proportion of high‐*rrn* taxa, which are preferentially recovered under nutrient‐rich, short‐term cultivation, whereas low‐*rrn* taxa are systematically underrepresented. By linking *rrn* distributions to cultivation success, we propose trait‐informed strategies for media design, incubation regimes, co‐culture approaches, and preservation methods. This framework provides a predictive, generalizable path to bridge sequencing and cultivation, facilitating biodiversity conservation, sustainable agriculture, and microbiome‐based therapeutics.

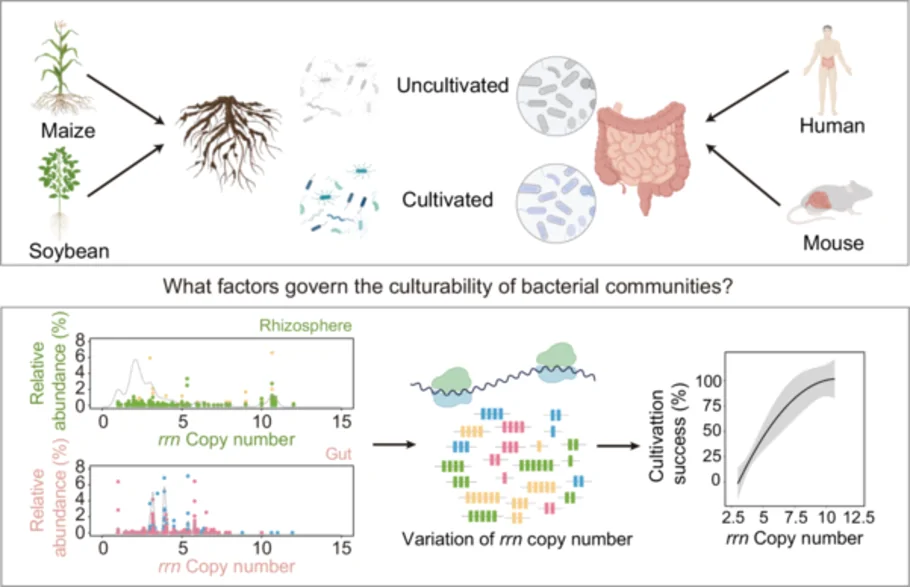

For over a century, microbiologists have faced the “great plate count anomaly”: microscopic counts of microbes far exceed colony‐forming units [1, 2]. Despite advances in sequencing, single‐cell genomics, and high‐throughput culturomics, most taxa remain uncultured, limiting functional validation, biobank development, and applied innovations in agriculture, medicine, and biotechnology [3, 4, 5]. Conventional methods—favoring copiotrophic generalists in nutrient‐rich, short‐term incubations—consistently miss slow‐growing oligotrophs, which often play key roles in ecosystem function [6, 7, 8]. To bridge this gap, we propose moving from empirical trial‐and‐error to a predictive, trait‐informed approach.

In macroecology, functional traits—quantitative measures of form, physiology, or behavior—help explain and are correlated with ecological patterns. In microbial ecology, the ribosomal RNA operon (*rrn*) copy number is a particularly informative genomic trait [9, 10, 11]. It reflects an organism's capacity for ribosome synthesis, influencing growth rate and nutrient response. Importantly, *rrn* copy number is stable within lineages, phylogenetically structured, and estimable from community sequencing data. We argue that *rrn* copy number can bridge genomic profiling and cultivation strategy design, enabling targeted approaches to recover underrepresented taxa.

Ribosome production capacity sets an upper limit on protein synthesis and maximum growth rate. Taxa with high *rrn* copy numbers can rapidly assemble ribosomes and exploit transient nutrient pulses, a hallmark of copiotrophic life strategies [9, 10, 11]. In contrast, low‐*rrn* taxa grow slowly and allocate resources toward efficiency, stress tolerance, or specialized metabolism typical of oligotrophy. These divergent strategies create predictable cultivation biases: high‐*rrn* taxa are readily recovered under nutrient‐rich, short‐term incubations, whereas low‐*rrn* taxa often require longer incubation, lower nutrient availability, or co‐culture approaches. By profiling *rrn* distributions in a target microbiome, researchers can anticipate these biases and tailor media composition, incubation regimes, and preservation methods accordingly. In this study, we extend *rrn*‐based trait insights from individual species to community‐level cultivation outcomes, and we propose a trait‐informed framework that links genomic profiling directly to actionable strategies for recovering uncultured microbial diversity.

## 1 COMMUNITY COMPOSITION AND TRAIT DISTRIBUTIONS IN THE RHIZOSPHERE

We profiled soybean (*Glycine max*) and maize (*Zea mays*) rhizosphere bacterial communities using 16S rRNA amplicon sequencing (Figures 1A,B, 2A), classifying ASVs into six rarity groups: always abundant taxa (AAT), conditionally rare or abundant taxa (CRAT), conditionally abundant taxa (CAT), moderate taxa (MT), conditionally rare taxa (CRT), and always rare taxa (ART) [12]. Dominant phyla included *Proteobacteria*, *Firmicutes*, and *Actinobacteria*, with crop‐specific differences: soybean favored *Proteobacteria* and *Firmicutes*, whereas maize showed higher Actinobacteria (Figure S1A,B,E,F).

**FIGURE 1:**
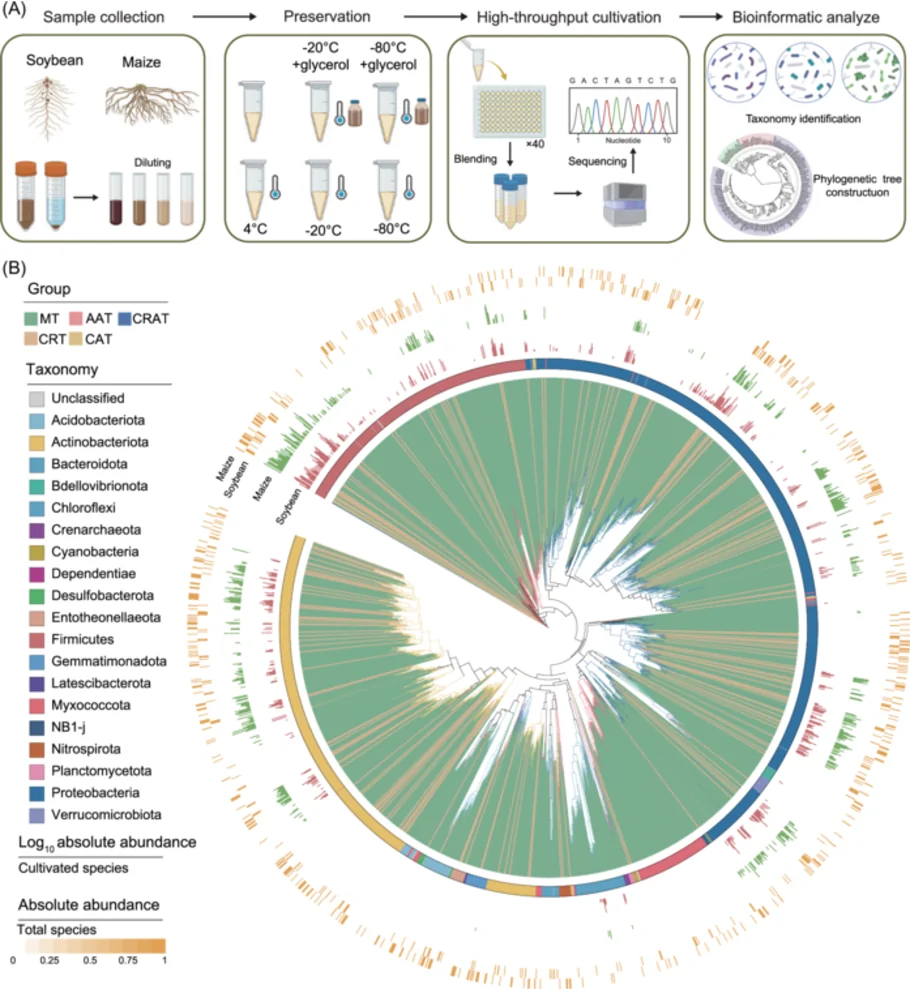
Distribution of different taxa in soybean and maize rhizosphere. (A) Overview of the experiment procedures. (B) Maximum‐likelihood phylogenetic trees were constructed from the top 2000 ASVs (out of 3431 total ASVs) identified from 16S rRNA gene amplicon sequencing of the rhizosphere microbiome, illustrating branching patterns that reflect shared ancestry and evolutionary divergence among taxa. The branches of the tree depict evolutionary paths, with longer branches indicating greater divergence and shorter branches indicating closer evolutionary relationships. The color of innermost clades indicates the distribution of taxa from different rarity group (MT: moderate taxa, AAT: always abundant taxa, CRT: conditionally rare taxa, CAT: conditionally abundant taxa, CRAT: conditionally rare or abundant taxa). The first ring indicates a different phylum. The red bar indicates the log10 relative abundance of cultivated ASVs in soybean rhizosphere, and the green bar indicates the abundance of cultivated ASVs in maize rhizosphere. The outermost heatmap indicates the relative abundance of all ASVs in soybean and maize rhizosphere.

**FIGURE 2:**
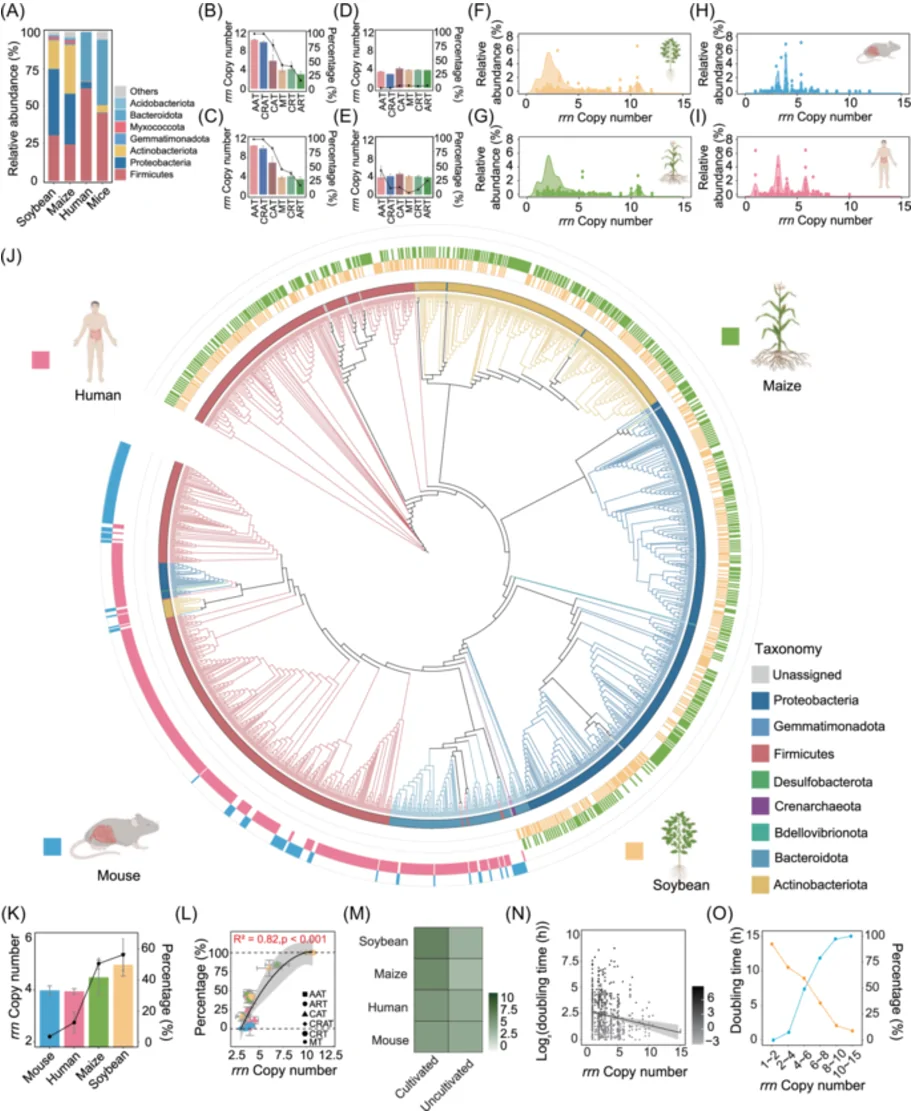
Life‐history strategies of culturable bacterial ASVs across different ecological niches for host–microbiome interactions. (A) Bacterial community composition in each ecological niche. Average ribosomal RNA operon (*rrn*) copy number and percentage of cultivated ASVs in the soybean rhizosphere (B), maize rhizosphere (C), human gut (D), and mouse gut (E) across different rarity groups. AAT: always abundant taxa, CRAT: conditionally rare or abundant taxa, CAT: conditionally abundant taxa, MT: moderate taxa, CRT: conditionally rare taxa, ART: always rare taxa. Scatter plots showing the distribution of individual ASVs by relative abundance and *rrn* copy number in the soybean rhizosphere (F), maize rhizosphere (G), human gut (H), and mouse gut (I). (J) Maximum likelihood phylogenetic tree of culturable ASVs (minimum absolute abundance ≥ 1), constructed from high‐throughput 16S rRNA gene sequences of rhizosphere and gut microbiomes. Branch lengths reflect evolutionary divergence. Concentric rings represent the environmental distribution of culturable ASVs: the innermost ring indicates taxonomic assignment at the phylum level; subsequent rings represent occurrence in the soybean rhizosphere, maize rhizosphere, human gut, and mouse gut, respectively. (K) Average *rrn* copy number and percentage of cultivated ASVs across each ecological niche, illustrating differences in life‐history strategies. (L) Relationship between the percentage of ASVs that were cultivable and their average *rrn* copy number, showing that taxa with higher *rrn* copy numbers were more frequently cultured. Blue: mouse, pink: human, green: maize, yellow: soybean. (M) Heatmap comparing the average *rrn* copy numbers between cultivated and uncultivated bacterial taxa across all rhizosphere and gut ecological niches. (N) Relationship between *rrn* copy number and doubling time (growth rate) for 700 bacterial and 190 archaeal species. (O) Relationship between *rrn* copy number and both doubling time (orange) and predicted cultivation success (blue).

High‐*rrn* copy numbers clustered in abundant groups (AAT, CRAT, CAT), while rare groups (ART, CRT) carried low *rrn* values (Figure 2B,C). This separation aligns with ecological strategies: abundant groups consisted of fast‐growing copiotrophs, whereas rare groups included slow‐growing specialists, many from *Acidobacteria* and *Verrucomicrobia*.

## 2 CULTIVATION PATTERNS AND THRESHOLD EFFECTS

High‐throughput cultivation in 10% TSB medium revealed a strong positive correlation between *rrn* copy number and bacterial cultivability (*R*² = 0.97 in soybean and *R*² = 0.99 in maize), corresponding to the proportion of ASVs successfully isolated from the rhizosphere of both crops (Figure S1D,H and Tables S1, S2). Beyond this threshold, additional *rrn* copies provided diminishing returns, likely reflecting physiological constraints unrelated to ribosome number [13]. At the phylum level, outcomes reflected this trend: *Proteobacteria* (generally high‐*rrn*) exhibited high cultivability; *Firmicutes* (very high *rrn*) dominated AAT groups; *Actinobacteria* (intermediate *rrn*) showed genus‐dependent variation (Figure 1B, Figure S1C,D,G,H).

## 3 PRESERVATION BIASES

Fresh samples captured the broadest diversity. Cold storage without cryoprotectant reduced Proteobacteria and enriched *Actinobacteria*, while glycerol partially restored *Proteobacteria* recovery. Low‐*rrn* taxa declined under all storage regimes, highlighting compounding effects of cultivation and preservation biases (Figure S2).

## 4 CROSS‐ECOSYSTEM COMPARISON: RHIZOSPHERE Versus GUT MICROBIOMES

We compared rhizosphere communities with human and mouse gut microbiomes as ecological benchmarks to evaluate *rrn*‐based trait distributions across ecosystems [14, 15, 16]. Clear ecosystem‐specific patterns emerged: rhizosphere communities exhibited higher mean *rrn* values (~6–8), with many taxa carrying more than nine copies, whereas gut communities had lower means (~3–5) and were dominated by oligotrophic lineages (Figure 2A–E).

These trait profiles were correlated with cultivation outcomes. Gut communities, enriched in low‐*rrn* taxa, displayed substantially lower cultivability under standard nutrient‐rich, aerobic conditions. Mouse gut communities, although exhibiting slightly higher *rrn* copy numbers than the human gut, remained considerably less cultivable than their rhizosphere counterparts (Figure 2F–K), particularly among abundant taxa (Figure S3). These differences reflect habitat‐specific selective pressures: rhizospheres are dynamic, nutrient‐pulsed, and structurally heterogeneous, favoring opportunistic, high‐*rrn* strategists; gut environments are chemically complex, host‐regulated, and relatively stable, selecting for efficient, syntrophic specialists.

## 5 CULTIVATION BIAS AND TRAIT‐BASED CORRECTIONS

Across all tested ecosystems, *rrn* copy number was positively correlated with cultivability, with cultivated taxa consistently exhibiting higher *rrn* copy numbers than their uncultured counterparts (Figure 2L,M). This predictable bias enriches fast‐growing copiotrophs while excluding low‐*rrn* oligotrophs (Figure 2N,O and Table S3). The omission of low‐*rrn* taxa is ecologically significant: slow‐growing genera such as *Variovorax*, *Ensifer*, and *Paenibacillus* contribute to nitrogen cycling, modulate phytohormones, and suppress plant disease [17, 18]. We propose *rrn*‐informed corrections to cultivation protocols: 1. Media design: Use oligotrophic or root exudate–based media to support low‐*rrn* taxa. Employ chemically defined media to reduce the dominance of fast growers. 2. Incubation regimes: Extend incubation periods for slow growers. Apply fluctuating nutrient conditions to mimic natural environments. Adjust temperature and oxygen levels to match ecological niches. 3. Co‐culture and in *situ* cultivation: Use diffusion chambers or iChips to preserve natural microbial interactions [19, 20]. Pair target taxa with helper strains for cross‐feeding or detoxification. 4. Preservation methods: Apply cryoprotectants and slow cooling to minimize selective losses. Minimize freeze–thaw cycles for sensitive, low‐*rrn* taxa. This trait‐based framework links *rrn* profiling to actionable cultivation strategies, providing a systematic approach to recover both abundant and elusive microbes.

Most microbial taxa remain uncultured—a challenge known as the “great plate count anomaly”—which constrains functional validation, microbial applications, and biodiversity conservation. Conventional cultivation methods disproportionately enrich fast‐growing, nutrient‐responsive taxa, leaving slow‐growing specialists underrepresented. Ribosomal RNA operon (*rrn*) copy number, however, provides a functional trait to anticipate these biases and guide corrective strategies. We propose *rrn*‐informed approaches to media formulation, incubation, and preservation that improve recovery of both abundant and elusive microbes. Integrating *rrn* profiling into isolation workflows enables researchers to systematically adjust protocols and capture underrepresented diversity. Although *rrn* is a strong predictor under the tested conditions, it should be considered alongside other genomic features—including genome size, GC content, and metabolic potential—for more comprehensive guidance. Future studies should probe causality by manipulating *rrn* copy number in model strains and by assessing how environmental and host contexts shape *rrn*–cultivability relationships. By linking trait‐based theory to practical cultivation, this framework narrows the gap between sequencing and experimentation, advancing efforts in sustainable agriculture, next‐generation probiotics, and microbial resource conservation.

## AUTHOR CONTRIBUTIONS

Bin Ni designed and supervised the study. Yuxiao Huang, Qingliu Wu, Pugang Yu, Hao Li, and Shengyue Tang performed the experiments. Shuangjiang Liu and Chang Liu provided the gut microbiome data. Fusuo Zhang contributed to conceptualization. Yuxiao Huang and Qingliu Wu drafted the manuscript. Bin Ni revised and finalized the manuscript. All authors approved the final version.

## CONFLICT OF INTEREST STATEMENT

The authors declare no conflicts of interest.

## ETHICS STATEMENT

The human gut microbiome study (APIMCAS2017049) was approved by the Research Ethics Committee of the Institute of Microbiology, Chinese Academy of Sciences. The mouse gut microbiome study (SYDWLL‐2024‐019) was approved by the Research Ethics Committee of the School of Life Sciences, Shandong University.

## Supporting information


**Figure S1.** Community composition and ecological traits of bacterial taxa with varying rarity levels in the rhizosphere of soybean and maize.
**Figure S2.** Composition and distribution of cultivated bacterial taxa under various preservation conditions from the rhizospheres of soybean and maize.
**Figure S3.** Comparison of life‐history strategies among bacterial ASVs across rarity groups and host habitats.

**Table S1.** The taxonomic information, *rrn* copy numbers, and group classifications of all isolates from the soybean rhizosphere.
**Table S2.** The taxonomic information, *rrn* copy numbers, and group classifications of all isolates from the maize rhizosphere.
**Table S3.** Data on doubling times for different species were obtained from a synthesis of bacterial and archaeal phenotypic trait data.

## Data Availability

Bacterial 16S data for both soybean and maize rhizosphere microbial community analysis in this paper were deposited in the Sequence Read Archive: https://www.ncbi.nlm.nih.gov/sra/?term=PRJNA1290512. Raw data of high‐throughput cultivation from both soybean and maize rhizosphere soil obtained in this study are available at https://www.ncbi.nlm.nih.gov/sra/?term=PRJNA1290521. The data and analysis scripts for this study are publicly archived in the GitHub repository: https://github.com/hyx3030268-ship-it/IMO8291224. Supplementary materials (methods, figures, tables, graphical abstract, slides, videos, Chinese translated version, and updated materials) may be found in the online DOI or iMeta Science http://www.imeta.science/imetaomics/.
