# Supplementary material for: Ribosomal RNA operon copy number: A trait‐informed framework to close the microbial cultivation gap
Source: IMetaOmics. 2025 Dec 25;3(1):e70071. doi: 10.1002/imo2.70071 (PMC13674445; doi:10.1002/imo2.70071)
Supplement: Supplementary file 1 [file IMO2-3-e70071-s002.docx]

**Supporting information to**

**Ribosomal RNA operon copy number: a trait-informed framework to close the microbial cultivation gap**

**Running title:** *rrn* Copy number as a guide to microbial cultivation

Yuxiao Huang^1#^, Qingliu Wu^1#^, Chang Liu^2^, Pugang Yu^1^, Hao Li^1^, Shengyue Tang^1^, Fusuo Zhang^1^, Shuangjiang Liu^2,3,4^, Bin Ni^1^*

^1^State Key Laboratory of Nutrient Use and Management, College of Resources and Environmental Sciences, National Academy of Agriculture Green Development, China Agricultural University, Beijing 100193, China

^2^State Key Laboratory of Microbial Technology, Shandong University, Qingdao 266237, Shandong, China

^3^State Key Laboratory of Microbial Diversity and Innovative Utilization, Institute of Microbiology, Chinese Academy of Sciences, Beijing 100101, China

^4^College of Life Sciences, University of Chinese Academy of Sciences, Beijing 100049, China

*Correspondence: [nibin@cau.edu.cn](mailto:nibin@cau.edu.cn) (Bin Ni)

^#^Yuxiao Huang and Qingliu Wu contributed equally to this study.

**Materials and methods**

**Experimental setup and soil sampling**

Pot experiments were conducted in the greenhouse, Beijing, China (40°1′45″ N, 116°17′11″ E). The soil used in the experiment was collected from the Quzhou Experimental Station, Hebei Province (36°51′48″ N, 115°0′59″ E), and is classified as salinized chao soil. Fresh topsoil was collected from the field, sieved through a 2-mm mesh to remove debris, air-dried, and stored at room temperature before use. Soybean (*Glycine max*) and maize (*Zea mays* L., cultivar 'Zhengdan 958') were used as the model crops. Briefly, seeds were surface-sterilized with 3% sodium hypochlorite for 20 minutes, followed by thorough rinsing with sterile distilled water. After germination on sterile seedling plates, ten-day-old healthy seedlings were transplanted into plastic pots (diameter: 15 cm), each containing 2 kg of prepared soil. Plants were grown at 28°C with natural light-dark cycles from June to October in the greenhouse [1].

Rhizosphere soil samples were collected at the maturity stage, ~90 days post-transplanting. Rhizosphere soil was defined as the soil tightly adhering to the roots. Roots were gently excavated, shaken to remove loosely attached soil, and placed in sterile 50 mL tubes. Rhizosphere soil was dislodged by vortexing root samples in sterile phosphate-buffered saline (PBS), and the resulting suspension was centrifuged at 3,500 × g for 15 minutes to pellet the rhizosphere fraction. Roots were then cut with sterile scissors and stored in sterile plastic bags. Bulk soil samples were collected at both jointing and maturity stages by pooling soil from pots without plants. All soil and root samples were flash-frozen in liquid nitrogen and stored at –80°C prior to further processing.

Experimental samples were stored under five different preservation conditions for 7 days: T4 (4 °C), T-20 (−20 °C), T-80 (−80 °C), T-20G (−20 °C with 40% glycerol), and T-80G (−80 °C with 40% glycerol). Fresh samples were immediately subjected to high-throughput bacterial isolation and cultivation (Mock). After 7 days, rhizosphere samples from all five preservation treatments underwent the same high-throughput bacterial isolation and cultivation process.

**DNA extraction, PCR amplification, and 16S rRNA gene sequencing**

DNA was extracted from 0.5 g of rhizosphere soil using the FastDNA™ Spin Kit for Soil (MP Biomedicals, USA), following the manufacturer’s protocol. DNA quality and concentration were assessed using a NanoDrop 2000 spectrophotometer (Thermo Fisher Scientific, USA). The bacterial 16S rRNA gene V5–V7 region was amplified using primers 799F (AACMGGATTAGATACCCKG) and 1193R (ACGTCATCCCCACCTTCC) [2]. PCR products were quantified with the Quant-iT™ PicoGreen™ dsDNA Assay Kit (Thermo Fisher Scientific), pooled in equimolar concentrations, and sequenced using the Illumina MiSeq PE300 platform (Illumina, USA) by Majorbio Bio-Pharm Technology Co. Ltd. (Shanghai, China). Raw sequencing reads were quality-checked with FastQC v0.10.1 and paired-end reads were further merged to create consensus sequences using the fastq_mergepairs command [3]. Sequences were demultiplexed, quality-filtered, and trimmed to remove barcodes and primers using QIIME2 [4,5]. Amplicon sequence variants (ASVs) were clustered at 97% sequence similarity using DADA2. Representative sequences were taxonomically classified against the SILVA 138 reference database using the classify.seqs command in Mothur v1.39.5 [6,7]. BLAST analysis was used to identify cultivated ASVs in both rhizosphere soil and gut samples. ASVs from the rhizosphere that showed > 98% sequence identity to the full-length 16S rRNA gene sequences of the corresponding isolated strains were retained as highly matched ASVs. A phylogenetic tree was then constructed using a constrained topology search in FastTree v2.1.11.

**High-throughput cultivation and bacterial isolation**

High-throughput cultivation of the rhizosphere plant microbiome was performed as previously described, with minor modifications [8−11]. Briefly, a series of preliminary experiments were conducted to determine the optimal dilution concentration (ODC) for high-throughput cultivation. Fresh root systems and rhizosphere soils were homogenized and serially diluted in sterile PBS to final dilutions of 222×, 666×, 2,000×, 6,000×, 18,000×, and 54,000×. The diluted suspensions were inoculated into 96-well cell culture plates (totaling 45 plates). After 7 days of incubation at 28°C, bacterial growth was assessed by turbidity. The dilution resulting in approximately 30% of wells showing growth was defined as the ODC and used in subsequent experiments. High-throughput cultivation was then carried out using 10% Tryptic Soy Broth (TSB), with dilutions prepared at 1×ODC, ODC, and 3×ODC. After 14 days of incubation, plates with approximately 30% turbidity were selected for further analysis. To evaluate the impact of storage conditions on cultivability, root and rhizosphere samples were subjected to five different storage treatments: 4°C, –20°C, –20°C with 40% glycerol, –80°C, and –80°C with 40% glycerol. After 7 days of storage, high-throughput cultivation was repeated. Following incubation, 10 µL from each well across 40 of the 96-well plates was pooled and transferred into 10 mL centrifuge tubes. These composite samples, representing both fresh and stored conditions, were subjected to 16S rRNA gene sequencing for bacterial community analysis, using the same protocols as previously described.

**Definition and classification of bacterial taxa**

All ASVs were categorized into six mutually exclusive groups based on their relative abundance, following Dai et al. (2016): (i) Always rare taxa (ART): ASVs with abundance ≤ 0.1% in all samples; (ii) Always abundant taxa (AAT): ASVs with abundance ≥ 1% in all samples; (iii) Moderate taxa (MT): ASVs with abundance between 0.1% and 1% in all samples; (iv) Conditionally rare taxa (CRT): ASVs with abundance＜1% in all samples and ≤ 0.1% in some samples; (v) Conditionally abundant taxa (CAT): ASVs with abundance＞0.1% in all samples and ≥ 1% in some samples, but never ≤ 0.1%; (vi) Conditionally rare or abundant taxa (CRAT): ASVs with abundance ranging from ≤ 0.1% (rare) to ≥ 1% (abundant) [12].

**Data collection for mouse and human gut microbiomes**

DNA sequences from 10,558 human gut bacterial isolates—representing 400 microbial species obtained from 239 fecal samples of healthy donors through large-scale cultivation—along with human gut 16S rRNA sequencing data, were collected from previous studies [13]. DNA sequences of 1,437 cultured bacterial isolates, representing 126 different species from the mouse gut microbiome, along with mouse gut 16S rRNA sequencing data, were also obtained from previously reported studies [14]. It should be noted that the cultivation conditions used in these gut microbiome studies differed from those employed in our work.

**Estimation of bacterial *rrn* copy number and growth rate**

All the statistical analysis was performed based on the R version 3.6.1 ([http://www.r-project.org](http://www.r-project.org/)). Bacterial *rrn* copy numbers were estimated for ASVs using the *rrn*DB database (v5.4; <https://rrndb.umms.med.umich.edu/>) [15]. Each ASV was hierarchically matched against the reference database, starting from the lowest available taxonomic rank. When child taxon matches were identified, the mean *rrn* copy number across all matched child taxa was assigned to the ASV. In the absence of child-level matches, higher-level taxa were considered, and the mean *rrn* copy number of the corresponding parent taxa was used. Bacterial growth rates, expressed as doubling times, were subsequently inferred from database-derived estimates [16]. A second-order polynomial regression model (y ~ poly (x, 2)) was fitted to examine the relationship between bacterial *rrn* copy numbers and cultivability. Model performance was assessed using the coefficient of determination (R²) and the *p*-value, which were annotated on the plot with the stat_poly_eq ( ) function from the “ggpmisc” package.

**Reference**

1. Li, Hao, Qingliu Wu, Pugang Yu, Bin Ni. 2024. “Complete genome sequence of *Achromobacter* sp. strain E1, an endophyte from *Zea mays* L. cultivar (Zheng dan 958).” *Microbiology Resource Announcements* 13: e00560−00524. https://doi.org/10.1128/mra.00560-24

2. Bulgarelli, Davide, Matthias Rott, Klaus Schlaeppi, Emiel Ver Loren van Themaat, Nahal Ahmadinejad, Federica Assenza, Philipp Rauf, et al. 2012. “Revealing structure and assembly cues for *Arabidopsis* root-inhabiting bacterial microbiota.” *Nature* 488: 91−95. https://doi.org/10.1038/nature11336

3. Edgar, Robert C. 2010. “Search and clustering orders of magnitude faster than BLAST.” *Bioinformatics* 26: 2460−2461. https://doi.org/10.1093/bioinformatics/btq461

4. Bolyen, Evan, Jai Ram Rideout, Matthew R. Dillon, Nicholas A. Bokulich, Christian C. Abnet, Gabriel A. Al-Ghalith, Harriet Alexander, et al. 2019. “Reproducible, interactive, scalable and extensible microbiome data science using QIIME 2.” *Nature Biotechnology* 37: 852−857. https://doi.org/10.1038/s41587-019-0209-9

5. Caporaso, J. Gregory, Justin Kuczynski, Jesse Stombaugh, Kyle Bittinger, Frederic D. Bushman, Elizabeth K. Costello, Noah Fierer, et al. 2010. “QIIME allows analysis of high-throughput community sequencing data.” *Nature Methods* 7: 335−336. https://doi.org/10.1038/nmeth.f.303

6. Quast, Christian, Elmar Pruesse, Pelin Yilmaz, Jan Gerken, Timmy Schweer, Pablo Yarza, Jörg Peplies, Frank Oliver Glöckner. 2013. “The SILVA ribosomal RNA gene database project: improved data processing and web-based tools.” *Nucleic Acids Research* 41: D590−D596. https://doi.org/10.1093/nar/gks1219

7. Schloss Patrick, D., L. Westcott Sarah, Thomas Ryabin, R. Hall Justine, Martin Hartmann, B. Hollister Emily, A. Lesniewski Ryan, et al. 2009. “Introducing mothur: open-source, platform-independent, community-supported software for describing and comparing microbial communities.” *Applied and Environmental Microbiology* 75: 7537−7541. https://doi.org/10.1128/AEM.01541-09

8. Zhang, Jingying, Yong-Xin Liu, Xiaoxuan Guo, Yuan Qin, Ruben Garrido-Oter, Paul Schulze-Lefert, Yang Bai. 2021. “High-throughput cultivation and identification of bacteria from the plant root microbiota.” *Nature Protocols* 16: 988−1012. https://doi.org/10.1038/s41596-020-00444-7

9. Bai, Yang, Daniel B. Müller, Girish Srinivas, Ruben Garrido-Oter, Eva Potthoff, Matthias Rott, Nina Dombrowski, et al. 2015. “Functional overlap of the *Arabidopsis* leaf and root microbiota.” *Nature* 528: 364−369. https://doi.org/10.1038/nature16192

10. Zhang, Jingying, Yong-Xin Liu, Na Zhang, Bin Hu, Tao Jin, Haoran Xu, Yuan Qin, et al. 2019. “NRT1.1B is associated with root microbiota composition and nitrogen use in field-grown rice.” *Nature Biotechnology* 37: 676−684. https://doi.org/10.1038/s41587-019-0104-4

11. Zhang, Jingying, Bing Wang, Haoran Xu, Weidong Liu, Jingwei Yu, Qiuxia Wang, Hong Yu, et al. 2025. “Root microbiota regulates tiller number in rice.” *Cell* 188: 3152−3166. https://doi.org/10.1016/j.cell.2025.03.033

12. Dai, Tianjiao, Yan Zhang, Yushi Tang, Yaohui Bai, Yile Tao, Bei Huang, Donghui Wen. 2016. “Identifying the key taxonomic categories that characterize microbial community diversity using full-scale classification: a case study of microbial communities in the sediments of Hangzhou Bay.” *FEMS Microbiology Ecology* 92: fiw150. https://doi.org/10.1093/femsec/fiw150

13. Liu, Chang, Mengxuan Du, Rexiding Abuduaini, Haiying Yu, Danhua Li, Yujing Wang, Nan Zhou, et al. 2021. “Enlightening the taxonomy darkness of human gut microbiomes with a cultured biobank.” *Microbiome* 9: 119. https://doi.org/10.1186/s40168-021-01064-3

14. Liu, Chang, Nan Zhou, Mengxuan Du, Yutong Sun, Kai Wang, Yujing Wang, Danhua Li, et al. 2020. “The mouse gut microbial biobank expands the coverage of cultured bacteria.” *Nature Communications* 11: 79. https://doi.org/10.1038/s41467-019-13836-5

15. Stoddard, Steven F., Byron J. Smith, Robert Hein, Benjamin R. K. Roller, Thomas M. Schmidt. 2015. “*rrn*DB: improved tools for interpreting rRNA gene abundance in bacteria and archaea and a new foundation for future development.” *Nucleic Acids Research* 43: D593−D598. https://doi.org/10.1093/nar/gku1201

16. Madin, Joshua S., Daniel A. Nielsen, Maria Brbic, Ross Corkrey, David Danko, Kyle Edwards, Martin K. M. Engqvist, et al. 2020. “A synthesis of bacterial and archaeal phenotypic trait data.” *Scientific Data* 7: 170. https://doi.org/10.1038/s41597-020-0497-4

**Figure S1.** **Community composition and ecological traits of bacterial taxa with varying rarity levels in the rhizosphere of soybean and maize.** (A) Community composition of bacterial taxa categorized by rarity group in the soybean rhizosphere. (B) Proportional distribution of rarity groups in the soybean rhizosphere. (C) Community composition of cultivated bacterial taxa by rarity group in the soybean rhizosphere. (D) Correlation between cultivation success (% of ASVs cultured) and average *rrn* copy number per rarity group in the soybean rhizosphere. (E) Community composition of bacterial taxa categorized by rarity group in the maize rhizosphere. (F) Proportional distribution of these rarity groups in the maize rhizosphere. (G) Community composition of cultivated bacterial taxa by rarity group in the maize rhizosphere. (H) Correlation between cultivation success (% of ASVs cultured) and average *rrn* copy number per rarity group in the maize rhizosphere.

**Figure S2. Composition and distribution of cultivated bacterial taxa under various preservation conditions from the rhizospheres of soybean and maize.** (A) Phylogenetic tree of cultivated taxa from the soybean rhizosphere. The inner circle indicates taxonomic classification at the phylum level. The outer bar chart displays the log_₂_-transformed relative abundance of each ASV under six preservation treatments: Mock, T4, T-20, T-20G, T-80, and T-80G (in order). (B) Pie charts showing the relative proportions of bacterial phyla under each of the six sample preservation conditions in the soybean rhizosphere. (C) Ternary plot illustrating the enrichment patterns of bacterial phyla across the preservation treatments in the soybean rhizosphere. (D) Bar plots depicting the percentage of cultivable bacterial taxa recovered under each preservation condition in the soybean rhizosphere. (E) Stacked bar plots showing the relative abundance of bacterial phyla across the six preservation treatments in the soybean rhizosphere. (F) Phylogenetic tree of cultivated taxa from the maize rhizosphere, with the inner circle indicating phylum-level classification. The outer bar chart shows the relative abundance of each ASV under the six preservation conditions (Mock, T4, T-20, T-20G, T-80, T-80G). (G) Pie charts displaying the distribution of bacterial phyla across preservation conditions in the maize rhizosphere. (H) Ternary plot illustrating enrichment of bacterial phyla under different preservation conditions in the maize rhizosphere. (I) Bar plots showing the percentage of cultivated bacterial taxa (ASV level) under each preservation condition in the maize rhizosphere. (J) Stacked bar plots of the relative abundance of bacterial phyla across all preservation treatments in the maize rhizosphere.

**Figure S3. Comparison of life-history strategies among bacterial ASVs across rarity groups and host habitats.** Scatter plots show ASV distributions by rarity group, relative abundance, and *rrn* copy number in: soybean rhizosphere (A), maize rhizosphere (B), mouse gut (C), and human gut (D). AT: abundant taxa; MT: moderate taxa; RT: rare taxa.

**Table S1. The taxonomic information, *rrn* copy numbers, and group classifications of all isolates from the soybean rhizosphere.**

**Table S2. The taxonomic information, *rrn* copy numbers, and group classifications of all isolates from the maize rhizosphere.**

**Table S3. Data on doubling times for different species were obtained from a synthesis of bacterial and archaeal phenotypic trait data.**
